# Altitudinal gradients shaping tree diversity and regeneration dynamics in mountainous ecosystems

**DOI:** 10.1186/s12870-025-06677-6

**Published:** 2025-05-16

**Authors:** Syed Waseem Gillani, Mushtaq Ahmad, Muhammad Manzoor, Muhammad Waheed, Abeer Al-Andal, Beatrice Ambo Fonge

**Affiliations:** 1 Department of Plant Sciences, Quaid-i-Azam University, Islamabad, 45320 Pakistan; 2 Department of Botany, Faculty of Life Science, University of Okara, Okara, 56130 Pakistan; 3 Department of Biology, College of Science, King Khalid University, Abha, 61413 Saudi Arabia; 4 Department of Plant Science, University of Buea, PO BOX 63, Fako, Buea, Division South West Region, Cameroon

**Keywords:** Anthropogenic pressure, Climate change, Forest management, Kashmir Himalayas, Regeneration

## Abstract

**Background:**

Himalayan forests are crucial for ecological roles but face threats from natural and human factors. This study examines tree diversity, regeneration patterns, and human-induced threats in the Kashmir Western Himalayas. We use indices and multivariate analysis to investigate species richness, composition shifts, and the impact of ongoing anthropogenic threats on forest ecosystems.

**Methods:**

Vegetation sampling was conducted at 45 sites in the Kashmir Himalayas, at elevations of 600 to 3600 m. Data were collected using quadrat methods. To investigate species diversity, composition, and human influences, statistical analyses such as Shannon and Simpson indices, Non-metric multidimensional scaling (nMDS), Principal Component Analysis (PCA), Canonical Correspondence Analysis (CCA), and regression models were performed using R software and OriginPro.

**Results:**

A total of 33 tree species were recorded in different ecological zones: the temperate zone had the most species (22), followed by the subtropical zone (16) and the subalpine zone (7). The temperate zone had the highest Shannon index (2.15 ± 0.24) and Simpson index (0.86 ± 0.03), while the subalpine zone had the lowest Simpson index (0.48 ± 0.20). The subtropical zone had the highest evenness index (0.95 ± 0.04). PCA showed that PC1 explained 37.2% of the variation and PC2 explained 14.9%. Human-induced disturbances were significant drivers of species composition shifts, particularly in the subtropical and temperate zones, accounting for 11% of the variation. *Picea smithiana* had the highest density in the temperate zone (615.62 individuals per hectare). The regression analysis indicated a quadratic relationship between tree density and DBH (R² values from 0.66641 to 0.92089). Regeneration patterns varied: *Pinus roxburghii* had high seedling density in the subtropical zone, while *Abies pindrow* and *Pinus wallichiana* regenerated well in the temperate zone, and recruitment was limited in the subalpine zone.

**Conclusion:**

Elevation significantly influences on tree diversity and regeneration patterns, while human-induced factors shape species composition. Anthropogenic activities notably affect tree diversity, especially at lower elevations, thereby threatening ecosystem resilience. This study emphasizes the necessity of sustainable forest management practices to mitigate human impacts and promote forest regeneration, particularly in subtropical and temperate zones.

**Supplementary Information:**

The online version contains supplementary material available at 10.1186/s12870-025-06677-6.

## Introduction

The Himalayas are one of the world’s most diversified and distinct ecosystems, with many forest types arising from major altitudinal and climatic variations, ranging from lowlands to alpine summits [1, 2]. Distribution of vegetation is directly related to altitude, which determines the temperature gradient, a critical component in establishing vegetation types and determining their diversity [3–6]. The southwest monsoon, which originates in the Bay of Bengal and is intercepted by the eastern Himalayas, brings most precipitation to the region, while the western regions become comparatively drier due to less rainfall [7]. Moisture gradients significantly influence the distribution and diversity of regional vegetation. Biodiversity is greater in the eastern regions, where the tree line reaches 4000 m, compared to the drier and less diverse western areas, where it descends to 3300 m [4, 8]. The Himalayas are complex, ever-changing ecosystems that provide a variety of ecosystem services [9]. The Kashmir Himalaya, located on the northwestern edge of the Himalayan biodiversity hotspot, is home to rich floristic diversity. The elevation gradient in this region supports a wide range of flora, from tropical forests to alpine meadows, encompassing an exceptionally broad spectrum of elevation and ecological zones [5, 10]. Studying tree dynamics, particularly their distribution across elevation gradients, is essential for biodiversity conservation and understanding ecological processes [2, 11].

Mountain ecosystems play an important role in shaping mountain biodiversity hotspots, which was recognized by the Convention on Biological Diversity (CBD) as early as 2004. Furthermore, the 2030 Agenda for Sustainable Development prioritizes the conservation of mountainous biodiversity, as stated in Sustainable Development Goal (SDG) 15, Target 4 [12]. Mountain regions are biodiversity hotspots worldwide [13, 14] and provide essential ecosystem services and products that sustain life. Consequently, protecting existing natural forests and incorporating tree planting into human-altered ecosystems have been recognized as effective nature-based solutions for addressing the crises of climate change and biodiversity loss [15]. To effectively restore degraded forest ecosystems, it is crucial to understand the composition, structure, diversity, and potential regeneration of tree species in natural forests [16].

Anthropogenic pressures, including deforestation, habitat degradation and fragmentation, overexploitation, invasive species, pollution, and global climate change, pose significant challenges to biodiversity [17, 18]. The extent, duration, and intensity of disturbances all affect ecosystem stability [19]. Assessing community structure, regeneration status, and disturbance levels is crucial for sustainable forest ecosystem management, as these factors influence the structure and composition of forest ecosystems [20]. Regeneration is vital for maintaining forest ecosystem dynamics and restoring degraded forest areas [21]. Additionally, the ability of native tree species to regenerate significantly affects the preservation of forest community structure [22]. Among various ecological processes, tree species regeneration plays a crucial role in sustaining their presence within a community across environmental conditions [23, 24]. Regeneration patterns serve as important indicators of forest health and can provide insights into a community’s future composition [25, 26]. Furthermore, the size-class distribution of forest trees is a key metric for analyzing forest structure and dynamics, frequently used to assess forest health, including species regeneration and recruitment [27, 28]. The regeneration ability of tree species is especially important in forests because it frequently influences the future composition of tree stands over time [29]. Specific habitat conditions are necessary for any species to regenerate, and the degree of these conditions significantly impacts the species’ global range [30]. Successful regeneration is important for forest sustainability [31]. Understanding the mechanisms that influence forest species regeneration is therefore important for both ecologists and forest managers [32].

Studying the regeneration of species is crucial for understanding the effects of human disturbances on community composition. Seedlings and saplings respond more rapidly to chronic, low-intensity disturbances than mature trees [33]. Forest regeneration, essential for maintaining ecological functions, ensures the survival of tree populations [34, 35]. Investigating how human activities influence this regeneration offers valuable insights into changes in forest composition, ecosystem processes, and the resilience of forests to environmental change [36–40]. A deeper understanding of these dynamics enhances our ability to predict long-term forest sustainability and directs conservation efforts aimed at reducing anthropogenic impacts on biodiversity and ecological stability. Kashmir’s Western Himalayas show significant elevation-driven variations in tree diversity and regeneration patterns across ecological zones [41]. There is a lack of integrated studies evaluating both natural and human-induced drivers of forest dynamics, particularly concerning sustainable forest management in this area. Existing studies often report specific zone ecology or focus on the ecological dynamics and distribution patterns of a single species, creating a research gap in detailed tree diversity in AJK [4, 42–45]. Therefore, this study sought to determine: (i) to assess elevation-based changes in tree diversity and composition across various ecological zones in the Kashmir Western Himalayas; (ii) to analyze tree species regeneration patterns across different ecological zones; (iii) to evaluate anthropogenic threats to tree diversity and regeneration; and (iv) to develop sustainable forest management strategies through ecological and human impact assessments.

## Materials and methods

### Study area

**Fig. 1:**
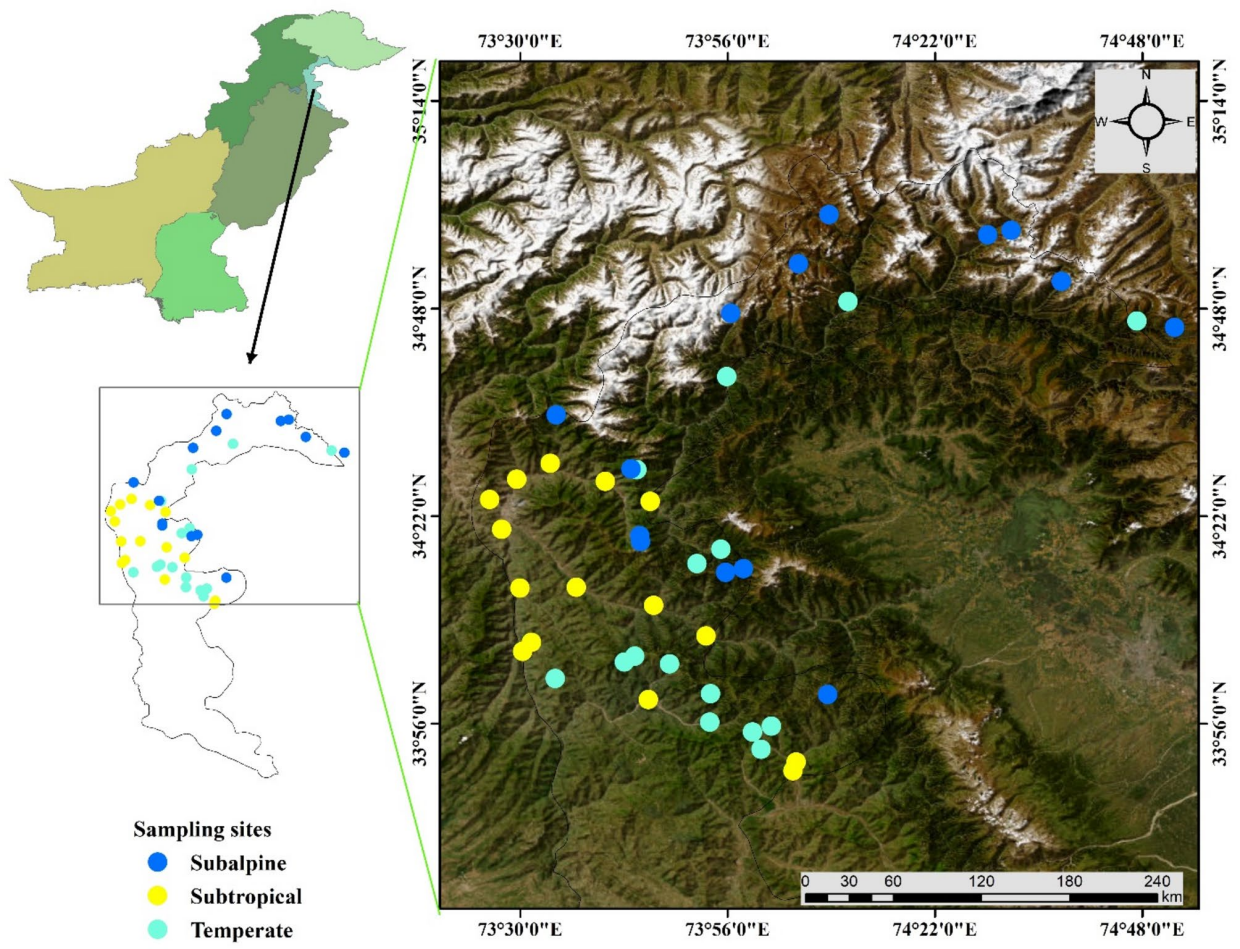
Study area map showing sampling sites across three ecological zones (Subtropical, Temperate, and Subalpine) in the Western Himalayas. Different colored dots represent sampling locations corresponding to the three zones: yellow for Subtropical, cyan for Temperate, and blue for Subalpine. The satellite imagery provides an overview of the topographical variation

The study area is located in the western Himalayan mountainous range of Azad Jammu and Kashmir, Pakistan, between 33°-35° North latitude and 73°-75° East longitude (Fig. 1) [1, 4]. The region’s geography is generally mountainous and rough, with deep valleys formed by various streams, rivers, and forested mountainsides that add to the picturesque beauty. AJK is an important biodiversity hotspot [42], with elevations ranging from 488 m in the southern Punjab Plains to 6,212 m in the north, containing varied agroclimatic conditions and habitats [46, 47]. This area has a typical monsoonal climate, with the majority of its rainfall falling between July and September due to the impact of the Himalayan Mountain systems. Notably, it receives about 70% more monsoon precipitation than nearby regions in Pakistan. The temperature in July ranged from 15 °C to 35 °C, whereas in January, it ranged from − 10 °C to 22 °C. The average annual precipitation in the region is 1,511 mm [45]. The relative humidity ranges between 58% and 84%. Snowfall occurs from November to March, with upper areas receiving up to 4 m and lower portions accumulating 3 to 6 feet. Winters are cold and long due to the heavy snowfall [42].

### Sampling and data collection

The quadrat method was used to sample tree species diversity along the elevation gradient in the region [41]. In the Kashmir Himalayan Region, 45 sites were set up at elevations ranging from 600 to 3600 m across different three ecological zones. For tree species distribution, diversity, and community structure, square quadrats measuring 31.6 m × 31.6 m (0.1 ha) were randomly placed at each site to capture the maximum diversity in the region. In each of the three ecological zones—subtropical, temperate, and subalpine—15 distinct sampling sites were selected. At each site, five quadrats were established for data collection, resulting in a total of 75 quadrats per ecological zone (15 sites × 5 quadrats per site). In total, across all three ecological zones, 225 quadrats were implemented (75 quadrats × 3 ecological zones). Additionally, we established quadrats for saplings measuring 5 m × 5 m, with a total of 10 quadrats at each site, and for seedlings measuring 1 m × 1 m, with a total of 20 quadrats at each site [48, 49]. The regeneration status of major tree species was assessed by comparing the proportional distribution of individuals at the seedling, sapling, and adult stages [50, 51]. Geographic factors, such as elevation, aspect, latitude, and longitude, were recorded using a GPS system, and the slope gradient was measured with a clinometer. A clinometer and a diameter tape were used to measure tree height and diameter at breast height (DBH), which is measured 1.3 m above the ground. Regeneration is considered good when the number of seedlings exceeds that of saplings, which in turn outnumber adult trees. Fair regeneration is defined as having seedlings equal to or greater than saplings, which are equal to or fewer than adult trees, or when a species persists solely in the sapling stage with no seedlings present [52]. A species is considered non-regenerating if it exists only in the tree stage and has no seedlings or saplings. In contrast, species found solely in the seedling stage without mature trees are classified as “new” species. Trees are defined as individuals with a diameter at breast height (DBH) larger than 30 cm (measured at 1.3 m above ground level), saplings have diameters ranging from over 11 cm to 30 cm, and seedlings have a diameter of less than 10 cm. All of the plants were identified with the help of local taxonomist Dr. Mushtaq Ahmad and verified by WorldFloraOnline (http://www.worldfloraonline.org), and the specimens have been preserved in the Herbarium of Pakistan (ISL) at Quaid-i-Azam University in Islamabad.

### Anthropogenic indicators

**Fig. 2:**
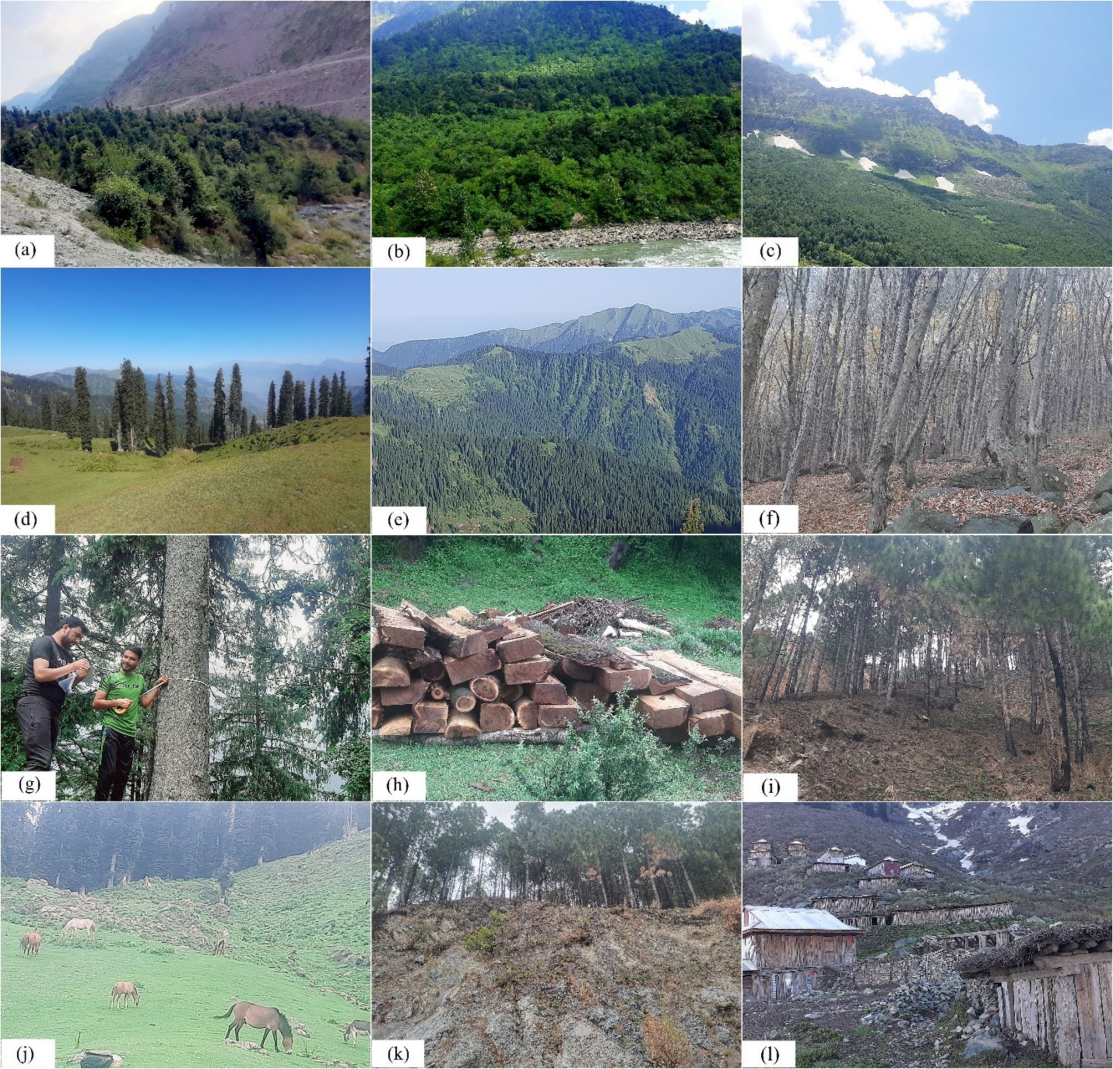
Filed photographs during data collection; (**a**) representing subtropical *Quercus* Forest, (**b**) temperate mix forest, (**c**) subalpine *Betula utilis* forests, (**d**) *Abies* dominant forest, (**e**) upper temperate and subalpine mix coniferous forest, (**f**) *Aesculus* dominant forest, (**g**) field measurements, (**h**) illegal deforestation in the study area, (**i**) fire affect visuals, (**j**) grazing, (**k**) soil erosion, (**l**) human settlement

Multiple anthropogenic disturbance factors, such as grazing, fire, deforestation, soil erosion, and human settlements, have been investigated and documented in the field (Fig. 2). Grazing intensity has been classified into three categories: overgrazed, moderate, and low, based on visual signs such as animal droppings, trampling effects, plant browsing evidence, and hoof imprints [4]. Soil erosion intensity was classified into three categories, such as severely eroded, moderately eroded, and low or no erosion, using key indicators like vegetation cover, exposed roots, slope gradient, and surface displacement based on the severity of erosion at each site. To assess deforestation, the number of tree stumps in each sample plot was counted as an indicator of logging activity [44]. Fire intensity was determined by examining burned vegetation cover within each sampling quadrat, which helped quantify the degree of fire impact in the research area (Fig. 2). Based on their distance from human settlements, which indicates the level of human activity and its effects on ecological processes, sites were divided into three categories: highly disturbed sites were those that were 0–1 km from settlements, moderately disturbed sites were those that were 1–2 km away, and least disturbed sites were those that were 2–3 km away from each data collection site. A three-point scale was used to determine the overall level of anthropogenic disturbance at each location. A score of 1 indicated minimal disturbance, 2 suggested moderate disturbance, and 3 indicated maximum. This visual evaluation methodology provided a systematic method for analyzing human-induced environmental stressors, providing consistency in data collection and interpretation across sampling sites.

### Data analysis

All statistical analyses and visualizations were performed using a combination of R software and OriginPro, depending on the specific analysis [53]. To visualize tree species distribution across ecological zones, a chord diagram was generated using the circlize package in R. This allowed for a clear representation of species overlap between the subtropical, temperate, and subalpine zones. Tree species diversity was assessed using Shannon, Simpson, Evenness, and Dominance indices, which were calculated for each ecological zone. These indices were represented using boxplots in OriginPro, and statistical differences among zones were evaluated using Tukey’s post hoc test following one-way ANOVA to determine significant differences in diversity across zones [54]. To examine differences in tree species composition among ecological zones, nMDS analysis was performed using the vegan package in R [55]. Bray-Curtis dissimilarity was used as the distance measure, and a stress value was calculated to assess the goodness of fit. Species composition and its relationship with ecological zones were further explored using Principal Component Analysis (PCA) in R, utilizing the Factoextra package [56]. The PCA biplot was generated to illustrate clustering patterns among ecological zones and to identify key species influencing each group. To assess the impact of environmental and anthropogenic factors on tree species composition, Canonical Correspondence Analysis (CCA) was conducted using CANOCO 5 [57]. The explanatory variables included human settlements, deforestation, erosion, fire, grazing, and slope, which were used to determine their relative influence on species distribution. The significance of these factors was tested using pseudo-F values and p-values. To evaluate the relationship between tree density (individuals/ha) and average DBH (cm) across ecological zones, quadratic regression models were applied in OriginPro. The dependent variable was tree density, while the independent variable was DBH. The quadratic model was selected based on visual trends in the data. All statistical analyses were conducted at a significance level of α = 0.05, and the results were interpreted accordingly.

## Results

### Composition of tree flora

**Fig. 3:**
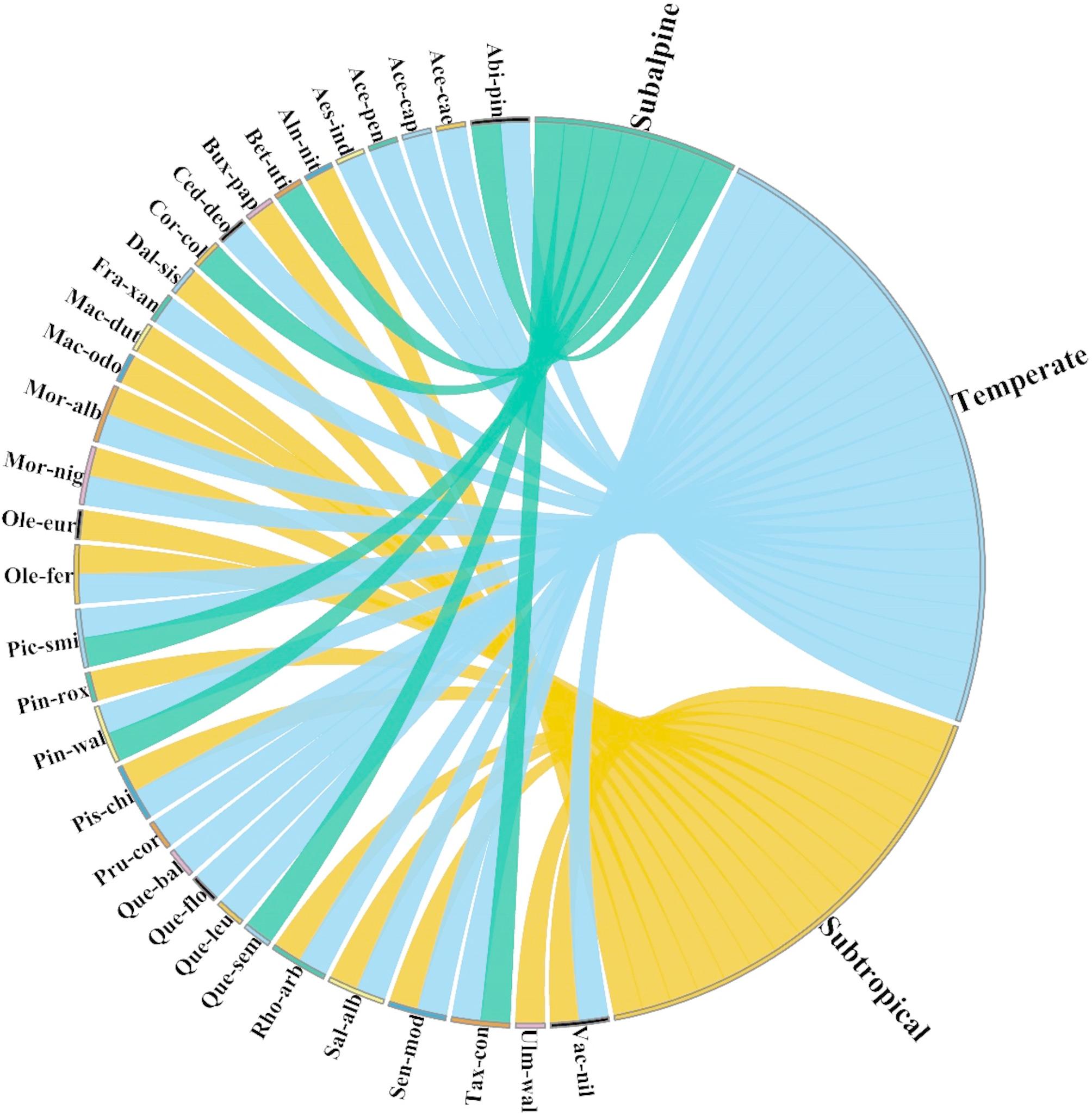
Chord diagram illustrating the distribution of tree species across three ecological zones (Subtropical, Temperate, and Subalpine) in the Western Himalayas. The connections represent species presence in multiple zones, with distinct colors indicating different zones. Species with links to multiple zones demonstrate ecological adaptability

A total of 33 tree species were recorded across three ecological zones in the western Himalayas: subtropical, temperate, and subalpine (Supplementary Table 1). Species richness varied among the zones, with the highest number of species observed in the temperate zone (22 species), followed by the subtropical zone (16 species). In contrast, the subalpine zone exhibited the lowest, with only seven species (Fig. 3). In the subtropical zone, tree included *Alnus nitida*,* Buxus papillosa*,* Dalbergia sissoo*,* Machilus duthiei*,* Machilus odoratissimus*,* Morus alba*,* Morus nigra*,* Olea europaea*,* Olea ferruginea*,* Pinus roxburghii*,* Pistacia chinensis*,* Rhododendron arboreum*,* Salix alba*,* Senegalia modesta*,* Ulmus wallichiana*, and *Vachellia nilotica*. The temperate zone included conifers such as *Abies pindrow*,* Cedrus deodara*,* Picea smithiana*,* Pinus wallichiana*, and *Taxus contorta*, along with several broad-leaved species like *Acer caesium*,* Acer cappadocicum*,* Acer pentapomicum*,* Aesculus indica*,* Fraxinus xanthoxyloides*,* Prunus cornuta*,* Quercus baloot*,* Quercus floribunda*,* Quercus leucotrichophora*, and *Rhododendron arboreum*. Some species, including *Morus alba*,* Morus nigra*,* Pistacia chinensis*,* Rhododendron arboreum*,* Salix alba*, and *Senegalia modesta*, were found in both subtropical and temperate zones, indicating their wide ecological adaptability. The subalpine zone exhibited the lowest tree diversity, with only seven species recorded. Key species included *Abies pindrow*,* Betula utilis*,* Corylus colurna*,* Picea smithiana*,* Pinus wallichiana*,* Quercus semecarpifolia*, and *Taxus contorta*. Unlike the other two zones, species overlap between the subalpine and subtropical zones was absent.

### Diversity pattern

**Fig. 4:**
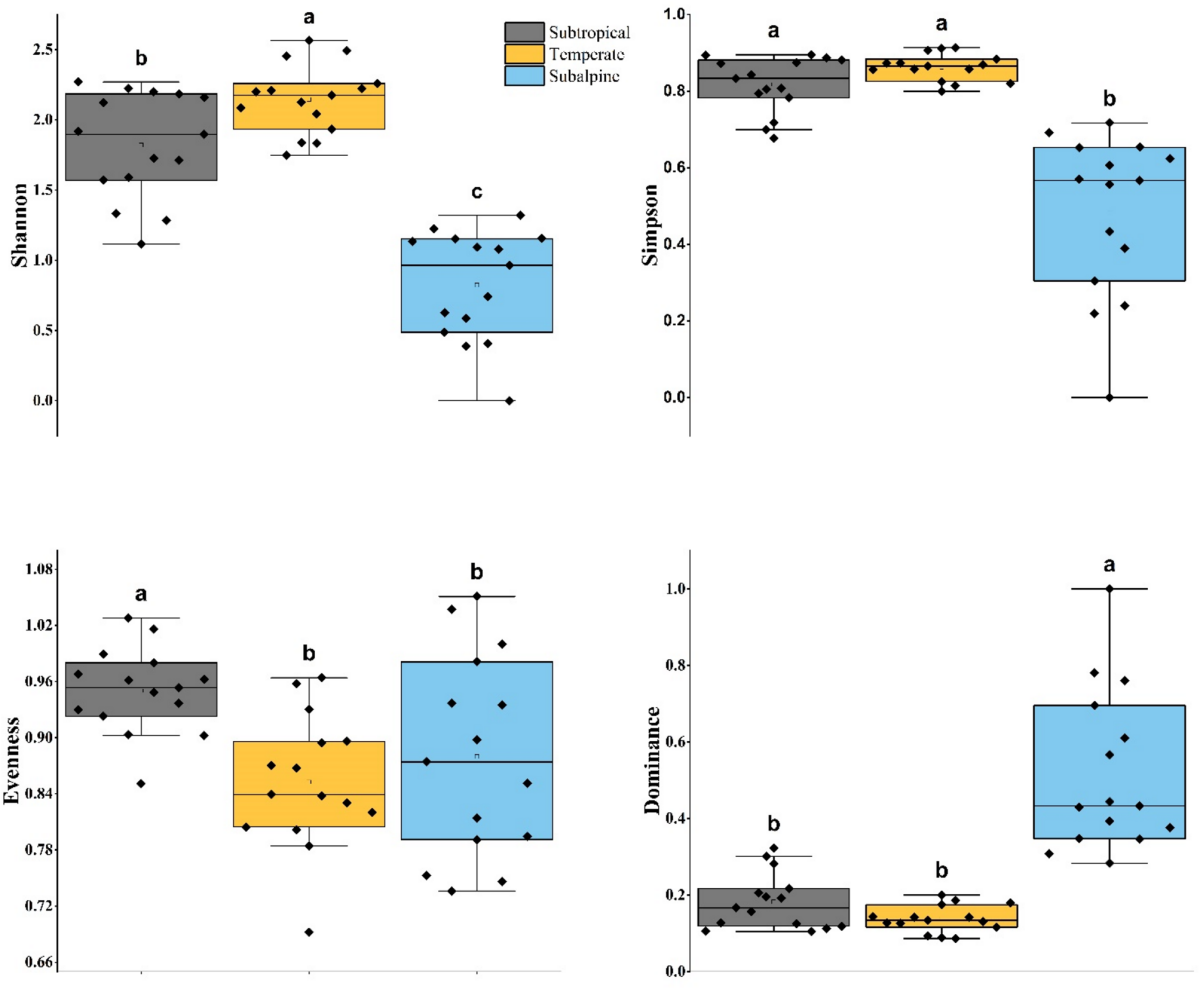
Boxplots showing variations in tree diversity indices (Shannon, Simpson, Evenness, and Dominance) across three ecological zones (Subtropical, Temperate, and Subalpine) in the Western Himalayas. Different letters above the boxplots indicate significant differences between zones based on Tukey’s post hoc test (*p* < 0.05)

The diversity indices varied significantly across the three ecological zones (subtropical, temperate, and subalpine) in the Western Himalayas. Tukey’s post hoc test was conducted to determine significant differences between zones. The Shannon diversity index was highest in the temperate zone (2.15 ± 0.24), followed by the subtropical zone (1.82 ± 0.38), and the lowest in the subalpine zone (0.82 ± 0.39). The differences were statistically significant, with the temperate zone exhibiting significantly higher diversity than both the subtropical (*p* = 0.035) and subalpine zones (*p* < 0.001). Additionally, the subtropical zone had significantly higher Shannon diversity than the subalpine zone (*p* < 0.001). The Simpson diversity index followed a similar pattern, with the highest value recorded in the temperate zone (0.86 ± 0.03), followed by the subtropical zone (0.82 ± 0.07), while the subalpine zone had the lowest Simpson index (0.48 ± 0.20). The post hoc test showed a significant difference between the subalpine and both the temperate (*p* < 0.001) and subtropical zones (*p* < 0.001), whereas there was no significant difference between the subtropical and temperate zones (n.s.). Species evenness was highest in the subtropical zone (0.95 ± 0.04), followed by the subalpine (0.87 ± 0.10) and temperate zones (0.85 ± 0.07). The subtropical zone showed significantly higher evenness than the temperate zone (*p* < 0.01), while the difference between the temperate and subalpine zones was marginally significant (*p* = 0.048). However, there was no significant difference between the subtropical and subalpine zones (n.s.) (Fig. 4). The dominance index was highest in the subalpine zone (0.51 ± 0.20), indicating a strong dominance of few species in this zone. The subtropical and temperate zones exhibited lower dominance values (0.18 ± 0.07 and 0.13 ± 0.03, respectively). The post hoc test revealed that dominance in the subalpine zone was significantly higher compared to both the temperate (*p* < 0.001) and subtropical zones (*p* < 0.001), while no significant difference was found between the subtropical and temperate zones (n.s.) (Table 1; Fig. 4).

**Table 1:** Summary of diversity indices (Shannon, Simpson, evenness, and Dominance) across three ecological zones (Subtropical, temperate, and Subalpine) in the Western himalaya. The table presents the mean values, standard deviation (SD), and statistical significance (p-values) from post hoc tests

Diversity indices	Zone	Mean	SD	Label
Shannon	Subtropical	1.82	0.38	*p* = 0.035
	Temperate	2.15	0.24	*p* < 0.001
	Subalpine	0.82	0.39	*p* < 0.001
Simpson	Subtropical	0.82	0.07	n.s.
	Temperate	0.86	0.03	*p* < 0.001
	Subalpine	0.48	0.2	*p* < 0.001
Evenness	Subtropical	0.95	0.04	*p* < 0.01
	Temperate	0.85	0.07	*p* = 0.048
	Subalpine	0.87	0.1	n.s.
Dominance	Subtropical	0.18	0.07	n.s.
	Temperate	0.13	0.03	*p* < 0.001
	Subalpine	0.51	0.2	*p* < 0.001

### Tree community differentiation across ecological zones

**Fig. 5:**
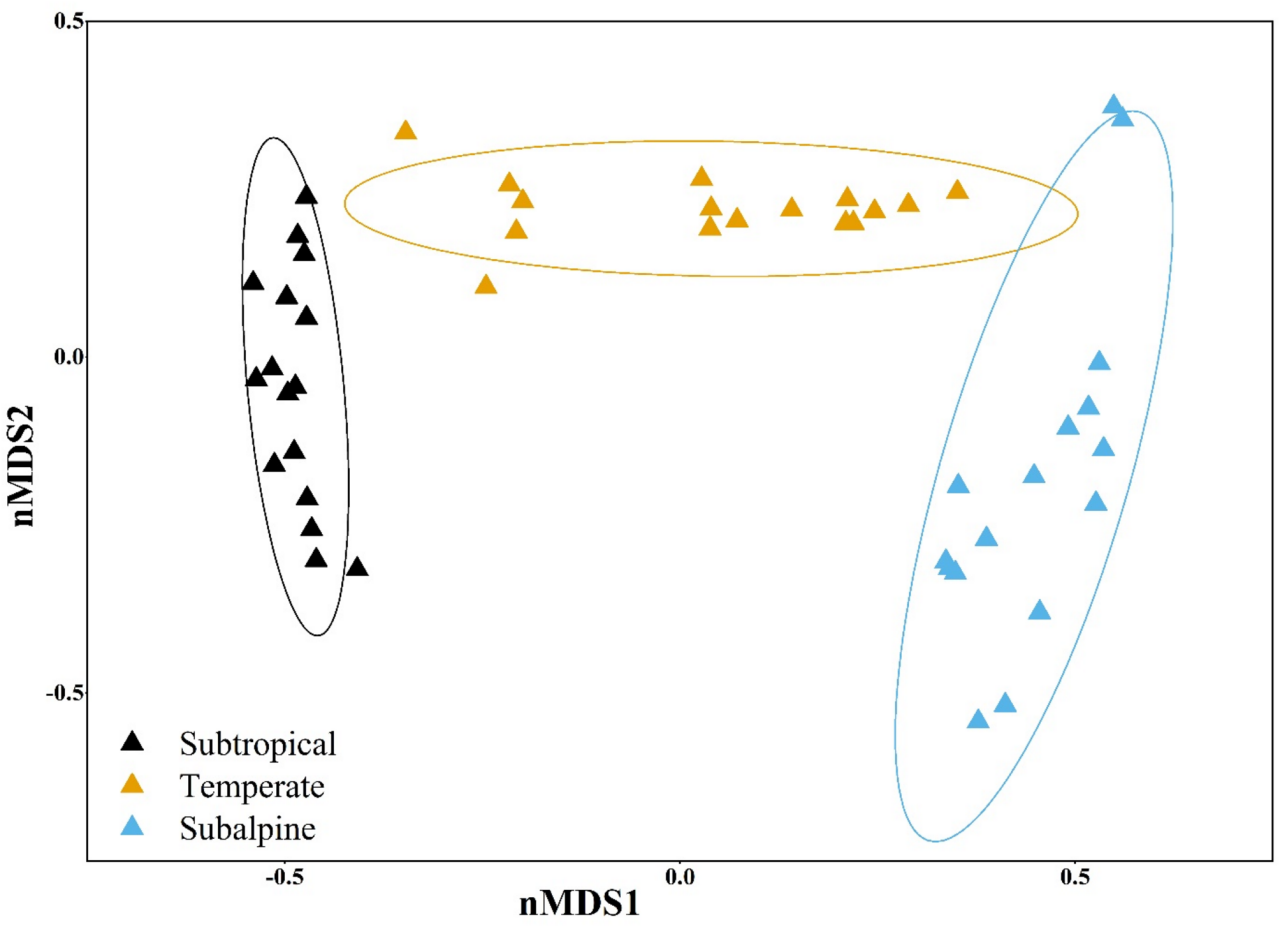
Non-metric multidimensional scaling ordination plot illustrating the differentiation of tree species composition across three ecological zones (Subtropical, Temperate, and Subalpine) in the Western Himalayas. Each triangle represents a sampling site, with different colors corresponding to ecological zones: black for Subtropical, yellow for Temperate, and blue for Subalpine. The ellipses indicate 95% confidence intervals for each zone. The distinct clustering of zones suggests significant variation in species composition, with minimal overlap, particularly between the subalpine and the other two zones

The non-metric multidimensional scaling (nMDS) analysis was conducted to visualize differences in tree species composition across the three ecological zones. The subtropical zone clusters on the left side of the ordination space, suggesting relatively homogenous species composition within this zone. The temperate zone is positioned centrally, forming a distinct cluster separate from both the subtropical and subalpine zones. The subalpine zone is distributed on the right side, forming a distinct and well-separated cluster. The clustering pattern suggests that species composition differs significantly across zones, with minimal overlap between them. The stress value of the nMDS analysis was 0.16, indicating a reasonably good ordination with an acceptable representation of the ecological distances between zones. The clear separation of the subalpine zone from the other two zones indicates a more unique species composition adapted to higher altitudes, while the subtropical and temperate zones show some level of differentiation but are relatively closer in composition compared to the subalpine zone (Fig. 5).

**Fig. 6:**
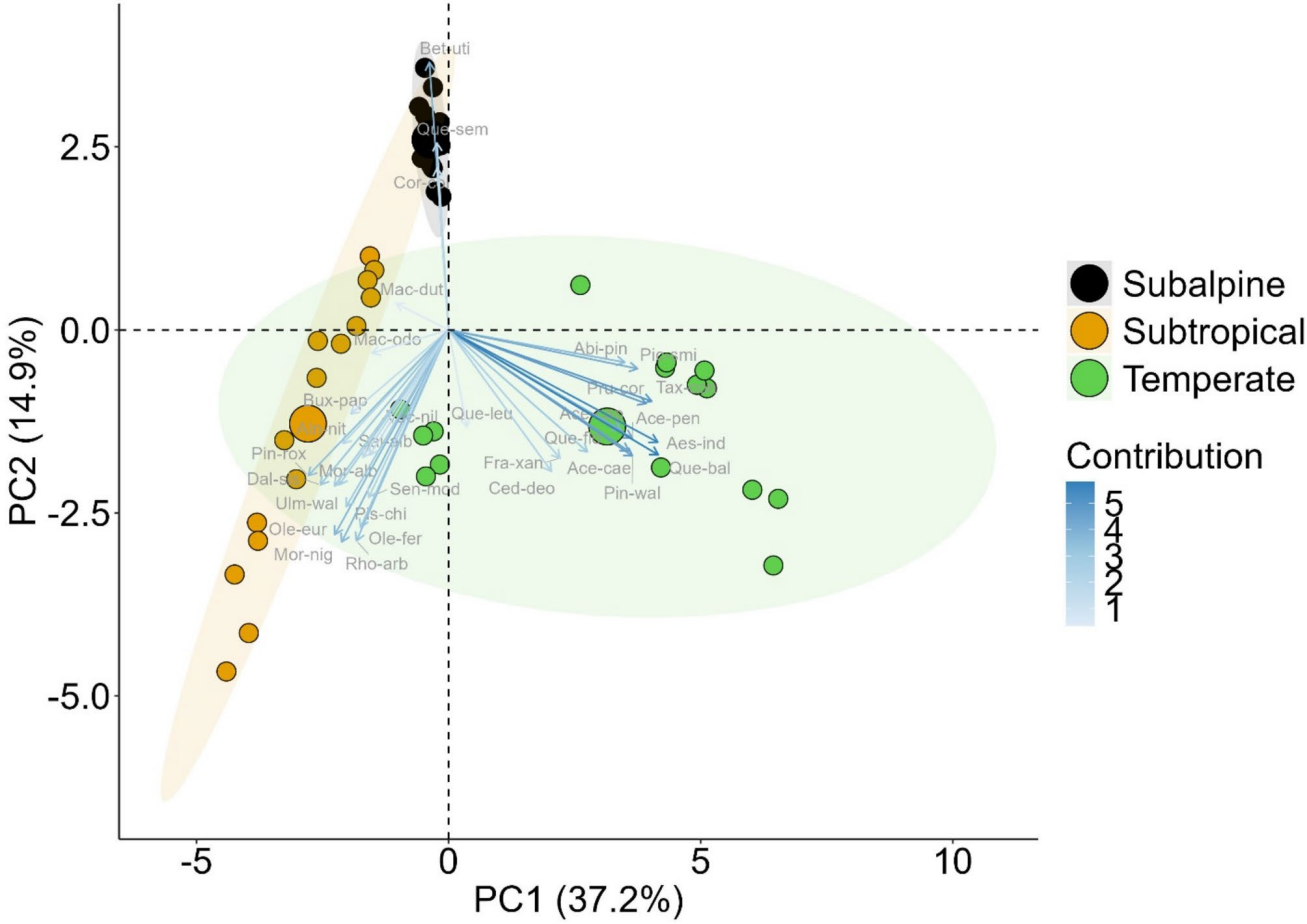
Principal Component Analysis biplot illustrating the variation in tree species composition across three ecological zones (Subtropical (Aln-nit = *Alnus nitida*, Bux-pap = *Buxus papillosa*, Dal-sis = *Dalbergia sissoo*, Mac-dut = *Machilus duthiei*, Mac-odo = *Machilus odoratissimus*, Mor-alb = *Morus alba*, Mor-nig = *Morus nigra*, Ole-eur = *Olea europaea*, Ole-fer = *Olea ferruginea*, Pin-rox = *Pinus roxburghii*, Pis-chi = *Pistacia chinensis*, Rho-arb = *Rhododendron arboreum*, Sal-alb = *Salix alba*, Sen-mod = *Senegalia modesta*, Ulm-wal = *Ulmus wallichiana*, Vac-nil = *Vachellia nilotica*), Temperate (Abi-pin = *Abies pindrow*, Ced-deo = *Cedrus deodara*, Pic-smi = *Picea smithiana*, Pin-wal = *Pinus wallichiana*, Tax-con = *Taxus contorta*, Ace-cae = *Acer caesium*, Ace-cap = *Acer cappadocicum*, Ace-pen = *Acer pentapomicum*, Aes-ind = *Aesculus indica*, Fra-xan = *Fraxinus xanthoxyloides*, Pru-cor = *Prunus cornuta*, Que-bal = *Quercus baloot*, Que-flo = *Quercus floribunda*, Que-leu = *Quercus* leucotrichophora), and Subalpine (Bet-uti = *Betula utilis*, Cor-col = *Corylus colurna*, Que-sem = *Quercus semecarpifolia*) in the Western Himalayas. Each point represents a sampling site, color-coded by ecological zone: orange for Subtropical, green for Temperate, and black for Subalpine. The percentage of variance explained by the first two principal components (PC1 = 37.2%, PC2 = 14.9%) is indicated on the axes. Species vectors show the contribution of individual species to site differentiation, with longer arrows representing stronger influences

The Principal Component Analysis was conducted to assess the variation in tree species composition across the three ecological zones. The first two principal components (PCs) explained a substantial proportion of the total variance in species distribution. PC1 accounted for 37.2% of the variance and primarily distinguished the temperate zone from the subtropical and subalpine zones. PC2 explained 14.9% of the variance, further differentiating the subalpine zone from the other two ecological zones (Fig. 6). The subtropical zone exhibited a well-defined cluster positioned on the negative side of PC1, reflecting its unique species composition. In contrast, the temperate zone formed a separate cluster on the positive side of PC1, highlighting the presence of species that are distinct from those found in the subtropical and subalpine regions. The subalpine zone was distinctly positioned along the positive axis of PC2, suggesting that species associated with this zone are adapted to high-altitude environmental conditions and are compositionally different from the species in the lower altitudes. Species contribution to the principal components varied, with certain taxa playing a significant role in differentiating ecological zones. Subalpine species such as *Betula utilis*,* Quercus semecarpifolia*, and *Corylus colurna* contributed strongly to PC2, reflecting their adaptation to colder and higher-altitude conditions. In contrast, temperate zone species, including *Abies pindrow*,* Pinus wallichiana*, and *Cedrus deodara*, had a high loading on PC1, emphasizing their ecological preference for mid-elevation regions. Subtropical species clustered towards the negative axis of PC1, indicating their distinct floristic composition compared to the temperate and subalpine zones. The PCA results demonstrate a clear compositional differentiation among the three ecological zones, reinforcing the findings of diversity indices and species distribution patterns. The strong separation of the subalpine zone suggests a highly specialized tree community adapted to extreme environmental conditions, whereas the subtropical and temperate zones, though distinct, show relatively closer floristic similarities (Fig. 6).

### Impact of anthropogenic factors on tree species composition

Canonical Correspondence Analysis (CCA) was conducted to assess the impact of environmental and anthropogenic factors on tree species composition across the three ecological zones. The total variation in species composition was 3.93122, with explanatory variables accounting for 28.0% of this variation, and an adjusted explained variation of 16.0%. The first two canonical axes explained a cumulative 22.78% of the total variation, with Axis 1 contributing 15.58% and Axis 2 contributing 7.2%. The high pseudo-canonical correlation values (0.8534 for Axis 1 and 0.6405 for Axis 2) suggest that the selected explanatory variables strongly influence the observed variation in tree species composition (Table 2). The subtropical zone is primarily associated with disturbances such as human settlements, deforestation, and fire, which are positioned along the negative side of Axis 1. The temperate zone appears to be moderately influenced by a mix of grazing and erosion, while the subalpine zone is primarily associated with slope and minimal human disturbances, indicating its relative isolation from direct anthropogenic impacts.

**Fig. 7:**
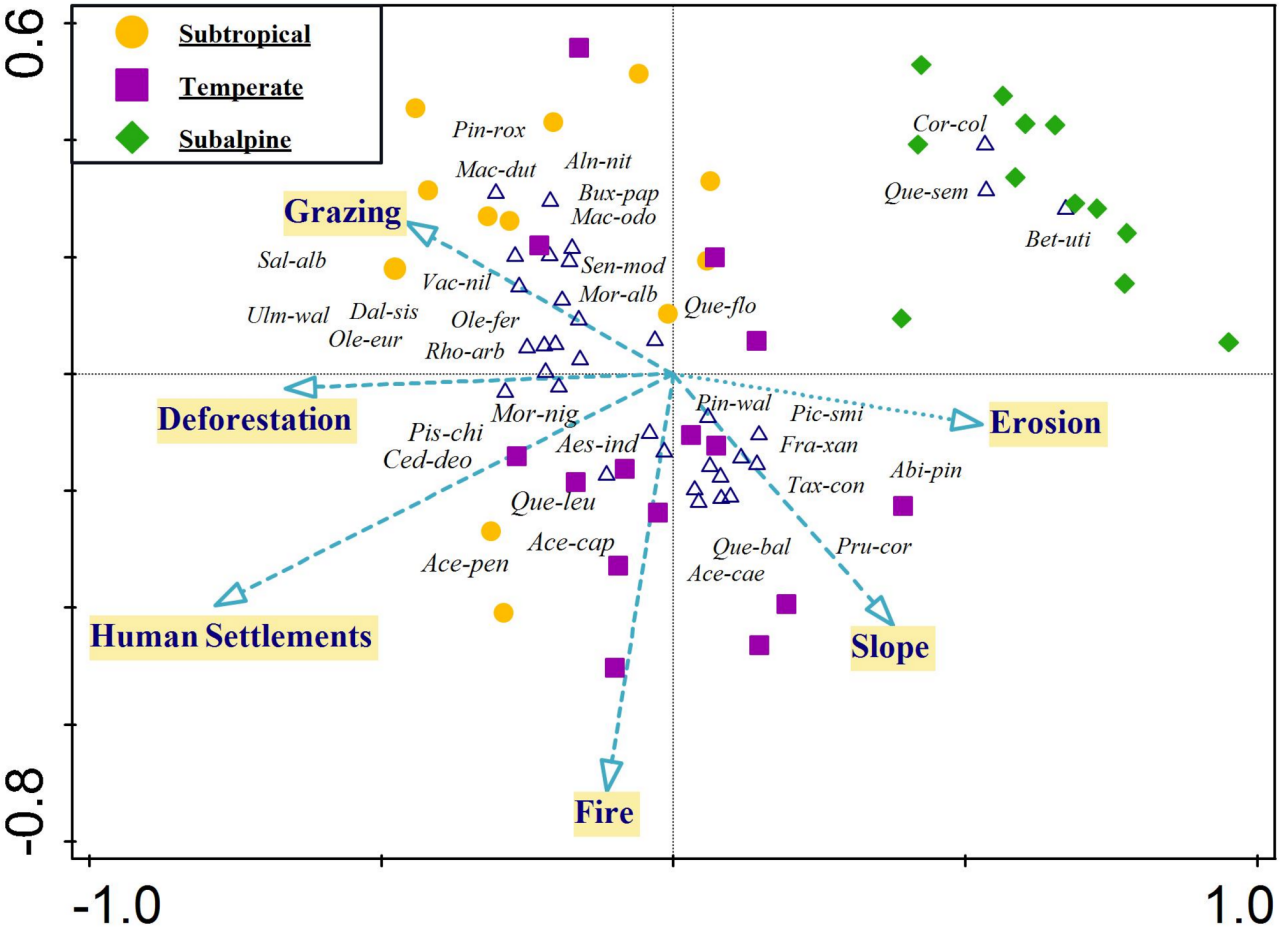
Canonical Correspondence Analysis ordination plot illustrating the influence of environmental and anthropogenic factors on tree species composition across three ecological zones (Subtropical, Temperate, and Subalpine) in the Western Himalaya. Each point represents a sampling site, color-coded by ecological zone: yellow for Subtropical, purple for Temperate, and green for Subalpine. Arrows indicate explanatory variables, with longer arrows representing stronger influences on species distribution

Among the individual threats, human settlements accounted for the highest percentage of explained variation (11%), with a significant effect (*p* = 0.002), indicating a strong influence of urbanization and land-use changes on tree species composition, particularly in the subtropical region. Deforestation also had a considerable impact, explaining 7.7% of the variation (*p* = 0.002), suggesting that logging and land clearance are major drivers of species distribution shifts. Erosion explained 5.1% of the variation (*p* = 0.016), highlighting the role of soil degradation and loss of vegetative cover in altering species patterns. Similarly, fire (4.6%, *p* = 0.046) and grazing (4.6%, *p* = 0.036) were significant contributors to species composition changes, particularly in the subtropical and temperate regions (Table 3). The slope was the least influential factor (4.2%, *p* = 0.064), indicating that topographical variation plays a role but is not as significant as direct anthropogenic pressures. The CCA results suggest that human-induced disturbances, particularly settlements, and deforestation, are the most significant drivers of tree species composition changes in the subtropical and temperate zones (Fig. 7). The subalpine zone remains less affected by these threats, with slope playing a more prominent role in structuring its vegetation.

**Table 2:** Summary of canonical correspondence analysis (**CCA**) results showing the eigenvalues, explained variation, pseudo-canonical correlation, and explained fitted variation across four axes

Statistic	Axis 1	Axis 2	Axis 3	Axis 4
Eigenvalues	0.6126	0.2828	0.0757	0.065
Explained variation (cumulative)	15.58	22.78	24.7	26.36
Pseudo-canonical correlation	0.8534	0.6405	0.6549	0.6727
Explained fitted variation (cumulative)	55.59	81.24	88.11	94.01

**Table 3:** Summary of canonical correspondence analysis results showing the impact of environmental and anthropogenic factors on tree species composition across three ecological zones in the Western himalaya. The table includes the percentage of explained variation, pseudo-F values, and significance (p-values) for each factor

Variable	Explains (%)	Pseudo-F	*p*-value
Human Settlements	11	5.1	0.002
Deforestation	7.7	3.4	0.002
Erosion	5.1	2.2	0.016
Fire	4.6	2	0.046
Grazing	4.6	2	0.036
Slope	4.2	1.8	0.064

### Tree density and DBH across ecological zones

**Fig. 8:**
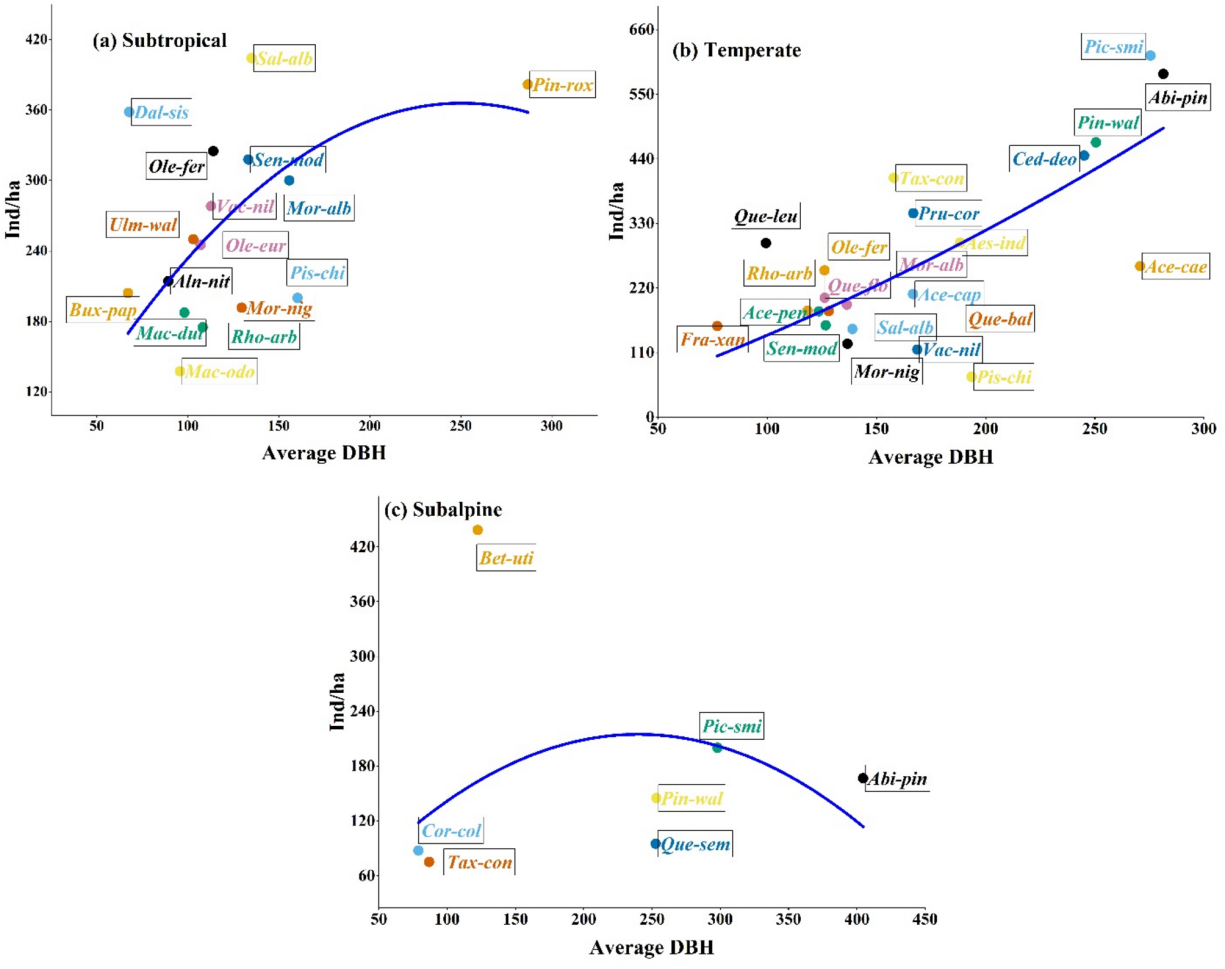
Relationship between tree density (individuals/ha) and average diameter at breast height (DBH) across three ecological zones in the Western Himalaya: (**a**) Subtropical, (**b**) Temperate, and (**c**) Subalpine. Each point represents a tree species, with labels indicating species codes. The fitted regression curves illustrate the quadratic relationship between DBH and density in each zone

The relationship between tree density (individuals/ha) and average diameter at breast height (DBH) was examined across three ecological zones: Subtropical, Temperate, and Subalpine in the Western Himalayas. In the subtropical zone, tree density ranged from 137.5 to 381.8 individuals/ha, with *Pinus roxburghii* exhibiting the highest density (381.8 individuals/ha) *and Macilus odoratissimus* showing the lowest (137.5 individuals/ha). The regression analysis showed a quadratic relationship between density and DBH (R² = 0.92089, Adjusted R² = 0.90959), suggesting that tree density decreases as DBH increases beyond a certain threshold. The negative B2 coefficient (-0.00582 ± 0.002) indicates a decline in density with increasing DBH, reflecting the prevalence of younger stands in high-density areas. In the temperate zone, species density ranged from 68.75 to 615.62 individuals/ha, with *Picea smithiana* showing the highest density (615.62 individuals/ha) and *Pistacia chinensis* the lowest (68.75 individuals/ha). The regression analysis also indicated a quadratic relationship between DBH and density (R² = 0.88668, Adjusted R² = 0.87535), with a positive B2 coefficient (0.00195 ± 0.00211), suggesting a moderate increase in density with DBH up to a certain limit before declining. The subalpine zone exhibited the lowest tree density, ranging from 75 to 438.46 individuals/ha. *Betula utilis* had the highest density (438.46 individuals/ha), while Taxus contorta had the lowest (75 individuals/ha). DBH values ranged from 79 cm (*Corylus colurna*) to 404.61 cm (*Abies pindrow*). Regression analysis indicated a weaker quadratic relationship compared to the other zones (R² = 0.66641, Adjusted R² = 0.53298), with a negative B2 coefficient (-0.00373 ± 0.00254) (Table 4). The relatively low explained variance suggests a more variable relationship between density and DBH (Fig. 8).

**Table 4:** Summary of quadratic regression analysis between tree density (individuals/ha) and average diameter at breast height (DBH) across three ecological zones (Subtropical, temperate, and Subalpine) in the Western himalaya

Zone	Residual Sum of Squares	*R*-Square (COD)	Adj. *R*-Square
Subtropical	93589.3	0.92089	0.90959
Temperate	232695.9	0.88668	0.87535
Subalpine	101196.4	0.66641	0.53298

**Fig. 9:**
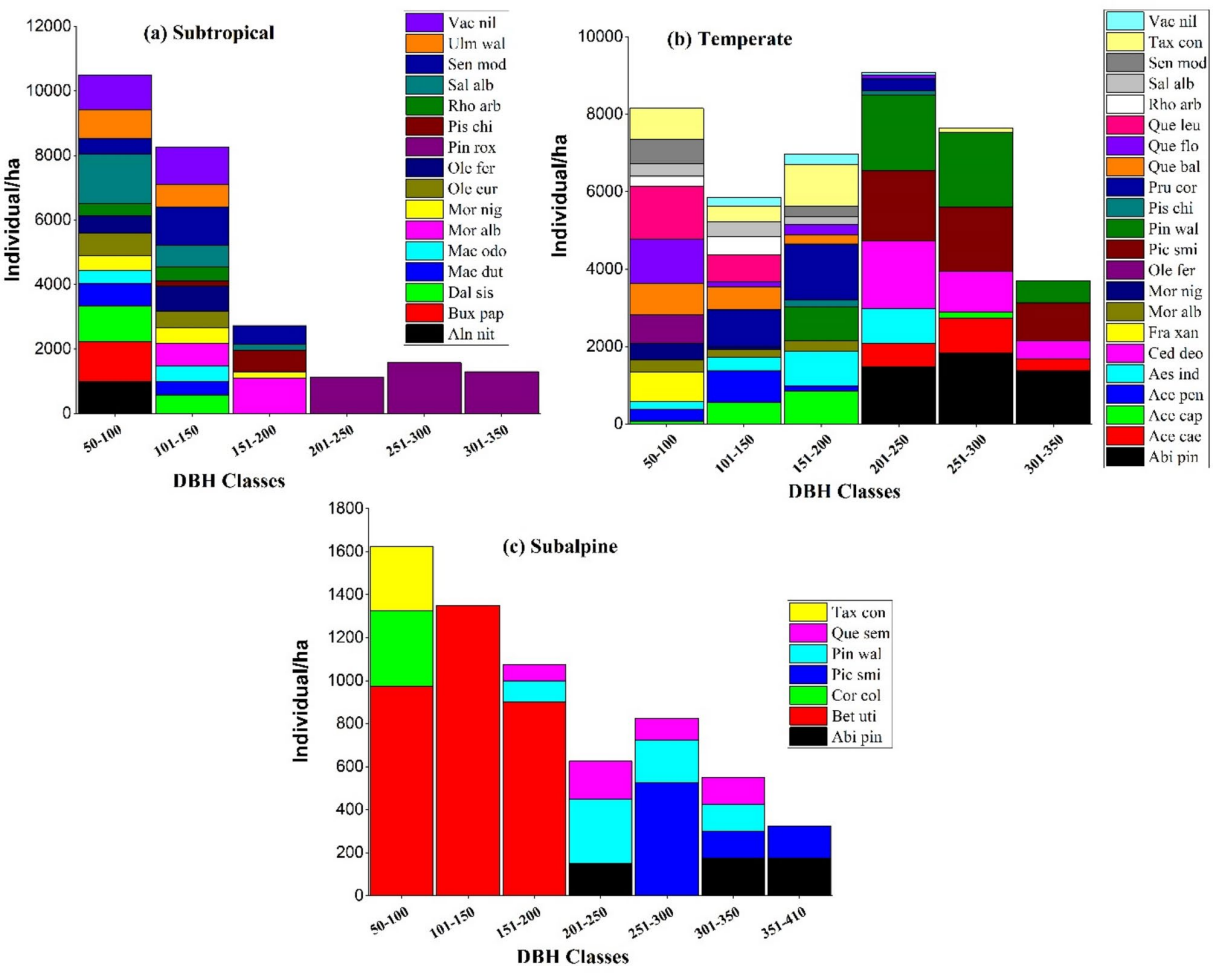
Diameter class distribution of tree species across ecological zones in the Western Himalayas: (**a**) Subtropical, (**b**) Temperate, and (**c**) Subalpine. Bars represent the number of individuals per hectare in each DBH class

The diameter class distribution of tree species across ecological zones showed distinct structural trends reflective of regeneration dynamics and disturbance regimes (Fig. 9). In the subtropical zone, a reverse J-shaped pattern was evident, with the highest tree densities in the 50–100 cm and 101–150 cm DBH classes, indicating robust regeneration dominated by species like *Pinus roxburghii*, *Dalbergia sissoo*, and *Alnus nitida*. The temperate zone exhibited a more uniform distribution across all DBH classes, with peaks at 151–200 cm and 201–250 cm, particularly of *Picea smithiana*, *Abies pindrow*, and *Cedrus deodara*. In contrast, the subalpine zone displayed a bell-shaped curve, with dominance in mid-diameter classes (101–200 cm) and fewer individuals in smaller and larger classes, likely due to environmental constraints affecting species such as *Betula utilis* and *Picea smithiana*.

### Regeneration patterns across ecological zones

**Fig. 10:**
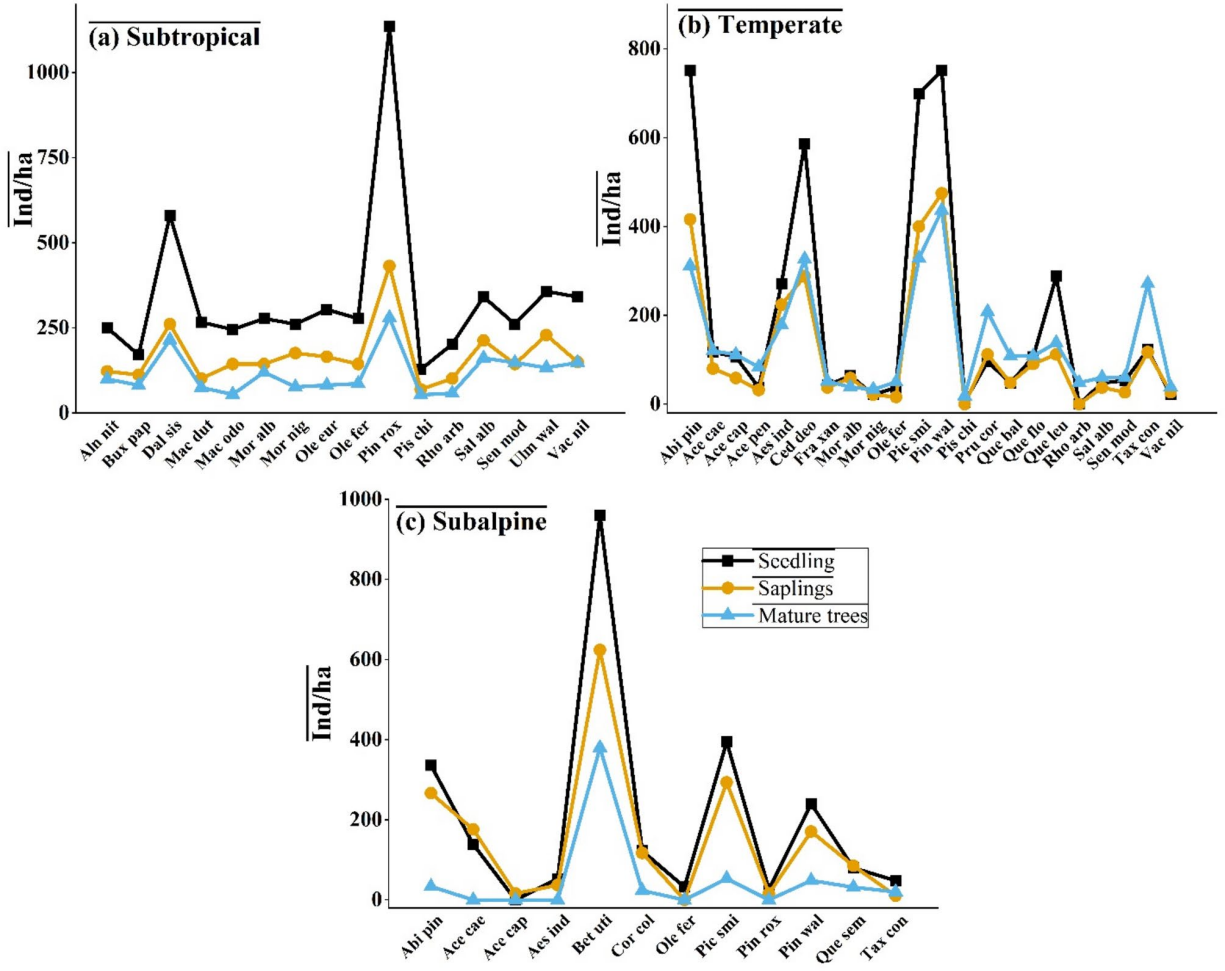
Regeneration status of tree species across three ecological zones in the Western Himalayas: (**a**) Subtropical, (**b**) Temperate, and (**c**) Subalpine. The graphs display the density of seedlings (black squares), saplings (orange circles), and mature trees (blue triangles) for each species in their respective zones

The regeneration status of tree species across the three ecological zones was assessed based on the density of seedlings, saplings, and mature trees. The data revealed significant variation in regeneration patterns among the zones. In the subtropical zone, tree regeneration exhibited considerable variability among species. *Pinus roxburghii* demonstrated the highest seedling density, indicating a strong regeneration potential. Other species, such as *Dalbergia sissoo*, *Alnus nitida*, *Salix alba*, and *Ulmus wallichiana*, also showed substantial seedling and sapling densities, suggesting successful recruitment. However, some species, such as *Macilus odoratissimus* and *Morus nigra*, had relatively low mature tree density despite higher seedling and sapling counts, indicating possible mortality or growth constraints (Fig. 10a).

In the temperate zone, species regeneration followed a more heterogeneous pattern. *Abies pindrow*, *Cedrus deodara*, *Picea smithiana*, and *Pinus wallichiana* exhibited strong regeneration, as evidenced by high densities of seedlings and saplings. However, some species, such as *Pistacia chinensis* and *Prunus cornuta*, showed low seedling establishment, which may indicate regeneration limitations due to environmental constraints or anthropogenic disturbances such as grazing and deforestation. *Quercus baloot* and *Quercus floribunda* displayed moderate seedling recruitment, though the gap between young and mature individuals suggests slow growth or high mortality rates (Fig. 10b).

Regeneration in the subalpine zone showed a distinct pattern, with a lower density of seedlings and saplings compared to mature trees in several species, indicating recruitment challenges. *Betula utilis* exhibited the highest seedling density, followed by *Picea smithiana* and *Abies pindrow*, reflecting strong natural regeneration in these species. However, species such as *Taxus contorta* and *Quercus semecarpifolia* showed disproportionately low seedling and sapling densities compared to mature trees, suggesting poor regeneration (Fig. 10c). The subtropical zone exhibited relatively higher regeneration potential for several species, with strong seedling and sapling recruitment. The temperate zone showed moderate regeneration success, with coniferous species displaying better recruitment than broadleaf species. In contrast, the subalpine zone exhibited a weaker regeneration potential, with limited seedling recruitment for several key species, raising concerns about long-term population sustainability.

## Discussion

Globally, Himalayan forests are diverse and feature complex ecosystems influenced by various factors, including elevation, climatic conditions, topography, and geographical extent [58]. Species diversity and richness are established indicators of community structure, and any changes can serve as measures of community dynamics [59]. We recorded 33 tree species across three ecological zones: subtropical, temperate, and subalpine. The temperate zone has the highest species richness (22 tree species), followed by the subtropical zone (16 tree species). The subalpine zone has the lowest tree diversity, with only seven species. The greater tree species diversity in the temperate zone is primarily due to its larger geographical area and extensive elevational range, as well as optimal conditions such as moderate rainfall, suitable temperatures, and well-adapted soil. These factors facilitate the growth of coniferous and broadleaf forests, leading to high species diversity [42]. In contrast, the subtropic zone exhibits moderate tree diversity values. The subalpine zone, on the other hand, showed a lower number of species due to extreme environmental conditions that limit the survival and regeneration patterns of tree species [60]. Furthermore, high snowfall, freezing temperatures, and a short growing season restrict tree growth, acting as a strong filter for only a limited number of species [4, 46]. Species overlap between the subalpine and subtropical zones is absent. Our results on tree species diversity are comparable to previous studies that also reported a similar number of tree species from the Himalayas [61, 62]. However, some studies reported low tree species diversity due to anthropogenic disturbances in their respective study areas, ranging from 18 to 28 species [63–65]. In contrast, other studies reported higher tree species diversity in mostly protected areas, ranging from 44 to 52 tree species [66, 67]. The significant variations in tree species diversity are attributed to various factors, including elevation, geographic location, biotic factors, and environmental conditions, which contribute to species diversity in different parts of Himalayas, as mountainous landscapes vary along the elevational gradient.

The nMDS analysis revealed significant differences in tree species composition across the three ecological zones. The clusters in the subtropical zone indicate a homogenous species composition, while the temperate zone is distinct from both. The subalpine zone’s unique cluster signifies a species composition specifically adapted to higher altitudes, whereas the subtropical and temperate zones show some differentiation. Elevation is a key geographical factor that defines the species distribution range, as it influences various climatic factors, including temperature, precipitation, and humidity, all of which play an essential role in the growth of tree species across different zones [68]. The dominant tree species recorded in the subtropical region include *Pinus roxburghii*,* Alnus nitida*,* Dalbergia sissoo*,* Machilus duthiei*,* Morus* spp., *Olea* spp., *Salix alba*,* Senegalia modesta*, and *Vachellia nilotica*. Our findings regarding the diversity of subtropical tree species align with those of Shaheen et al. [69] and Kharkwal et al. [70]. In the temperate zone, coniferous species such as *Abies pindrow*,* Cedrus deodara*,* Picea smithiana*,* Pinus wallichiana*, and *Taxus contorta*) are predominant, alongside broad-leaved species like *Acer caesium*,* Aesculus indica*,* Prunus cornuta*, and *Quercus* spp. Various researchers have consistently reported these species as dominant in the temperate forests of the Himalayas [41, 42, 44, 63, 71]. We observed tree species such as *Abies pindrow*,* Betula utilis*,* Corylus colurna*,* Picea smithiana*, and *Quercus semecarpifolia* as the dominant species in the subalpine zone. Various researchers have documented that these species are most common in two types of subalpine forests: forests that are only made up of *Betula* trees and forests that are mixed with coniferous species in the Himalayan timberline ecosystem [4, 72].

Tukey’s post hoc test revealed significant differences among the zones. The Shannon diversity index was highest in the temperate zone, followed by the subtropical zone. Species evenness was greatest in the subtropical zone, followed by the subalpine and temperate zones. The dominance index was highest in the subalpine zone, indicating a strong dominance of a few species. No significant difference was found between the subtropical and temperate zones. We recorded the highest number of tree species in the temperate zone, resulting in the highest Shannon diversity index. This is coupled with varying climatic and ecological conditions that support both pure coniferous and broad-leaved forests across wide elevational gradients in the region. Our findings align with various studies that have also reported the highest Shannon diversity index across different temperate forest ecosystems in the Himalayas [41, 44, 73]. In the present study, variations in Shannon’s diversity and species richness across various ecological zones are likely attributed to primary factors influencing diversity indices, including geographical variables, species count, and community structure [74, 75]. Furthermore, the subtropical zone experiences higher species evenness due to favorable climatic conditions, resource availability, longer growing periods, higher rainfall, and reduced environmental stress. These factors promote diverse plant communities where no single species dominates, resulting in a balanced distribution [43, 58, 69, 76]. In contrast, the subalpine zone, characterized by harsh environmental conditions, exhibits the highest dominance index of cold-adapted species, indicating a strong level of environmental filtering [4, 46]. The absence of a significant diversity difference between the subtropical and temperate zones may result from overlapping species pools and comparable disturbance levels.

Disturbance, whether natural or human-caused, significantly impacts forest structure and function. Human activity leads to extensive disruption, making it critical to understand how, when, and to what extent these disruptions affect ecosystem integrity and the ability of these ecosystems to provide essential services to society [61, 77, 78]. CCA indicated that human settlements, deforestation, and fire are the most significant drivers of changes in tree species composition in the subtropical and temperate zones. Anthropogenic factors significantly impact natural landscapes globally, leading to changes in climate, the environment, and natural resources. It poses a threat to these landscapes through fragmentation and other ecological issues [79]. Proximity to human settlements exacerbates these impacts and disrupts natural successional processes [80]. Together, these factors highlight the vulnerability of lower-elevation forests to anthropogenic pressures, necessitating targeted conservation strategies [81]. In contrast, the subalpine zone is less affected by these threats, with slope playing a more prominent role in shaping its vegetation. The degree of slope influences vegetation size, composition, and distribution by affecting wind speed, soil content, seed dispersal distance, and solar radiation intensity at higher elevations [82]. Additionally, steeper slopes may be better suited for deep-rooted tree species [83], which is the most probable reason for the dominance of a few species in the subalpine zone. The study found that these factors, along with soil degradation and loss of vegetative cover, are major drivers of shifts in species distribution. The subtropical zone is primarily impacted by human settlements, deforestation, and fire, while the temperate zone is moderately influenced by grazing and erosion. Overgrazing and soil erosion, along with the other aforementioned threats, are reported as major threats to the Himalayan temperate forest ecosystems by various researchers [44, 84, 85].

We examined the relationship between tree density and average diameter at breast height (DBH) in the Western Himalaya. Tree density and basal area are two key phytosociological characteristics that impact forest structure [41]. The subtropical zone exhibited the highest tree density, with *Pinus roxburghii* being the most prevalent species. Species diversity is significantly influenced by forest structure and composition, with high diversity often linked to more complex vertical structures [86]. The wide range of basal area values indicates a stable forest structure, consisting of both mature and young trees in the subtropical region of the study area [43, 87]. Significant variations in basal area are linked to site locations, resource availability, and disturbance history [88]. The subtropical zone exhibited higher density values, primarily because most sampled areas are located near the forest department, where deforestation activities are strictly prohibited. This regulation contributes to a more stable forest structure. However, some sites are significantly affected by anthropogenic activities and urban expansion, resulting in the removal of tree species in the region. A quadratic relationship was observed between density and DBH, indicating that as DBH increases, density declines. Forest stands tend to have higher densities of smaller-diameter trees, attributed to resource limitations and growth constraints, which reflect classical forest dynamics influenced by competition and environmental factors [89, 90]. In the temperate zone, species density varied from 68.75 to 615.62 individuals/ha, with *Picea smithiana* having the highest density. The observed differences in tree layers across ecological zones may be attributed to factors such as altitude, species composition, age, disturbances, and successional strategies. Our findings on the population structure of trees in the region are consistent with those from other forest stands in the Himalayas, which have reported similar results in their studies [41, 73, 76, 91, 92]. Additionally, the distribution of trees across various girth classes reflects how the forest utilizes functional and structural resources. Diameter distribution is commonly used to represent the population structure of forests [90]. The low density values in the temperate forest are attributed to rapid deforestation and anthropogenic activities, such as human encroachment, fire, fuelwood extraction, logging, and road construction [42, 44]. This situation highlights the urgent need for the conservation of the vital temperate forest ecosystem in the western Himalayas. The subalpine zone exhibited the lowest tree density, with *Betula utilis* demonstrating the highest density in that region. Due to harsh environmental conditions, such as high snowfall, freezing temperatures, strong winds, intense solar radiation, and a short growing season at higher elevations, *Betula utilis* is identified as an indicator species that is well adapted to these conditions and shows dominance throughout the subalpine zone [4]. Furthermore, some coniferous species were also observed in this zone, but only in limited numbers. Regression analysis revealed a weaker quadratic relationship in the subalpine zone, suggesting a more variable relationship between density and DBH.

Natural regeneration is a crucial process that ensures the persistence of forests and the establishment of new generations of trees [93]. The regeneration potential of a forest depends on the presence of sufficient seedlings, saplings, and mature trees, which can vary with changes in forest structure and physiographic conditions [41, 65]. We assessed the regeneration status of tree species to evaluate forest health status across three ecological zones, focusing on the density of seedling, sapling, and mature trees. The subtropical zone exhibited significant variability in regeneration patterns, with species like *Pinus roxburghii* demonstrating high seedling density and successful recruitment. However, some species exhibited low mature tree density, indicating potential mortality or growth constraints. The high seedling density and successful recruitment of *Pinus roxburghii* may be attributed to dry conditions [69]. In contrast, the low tree density observed in some species suggests that limitations in recruitment may be linked to variations in microclimatic conditions, soil properties, and anthropogenic disturbances that could affect regeneration success [64, 65, 76]. Subtropical forests may face decline as changes in regeneration patterns threaten their future survival due to a shortage of seedlings. Only *Pinus roxburghii* in the subtropical zone, with an equal distribution across all life stages, is expected to maintain dominance in the near future.

In the temperate zone, species like *Abies pindrow*,* Cedrus deodara*,* Picea smithiana*, and *Pinus wallichiana* showed strong regeneration. Significant regeneration of these coniferous species suggests the ecological adaptation, favorable environmental conditions, and disturbances regimes of these species across the temperate forest ecosystems in the region. These species are shade-tolerant, maintain a persistent seed bank, and efficiently capture light and moisture. Sufficient soil moisture, low disturbance levels, and minimized human pressures support seedling establishment and survival [42, 44, 75]. Similar regeneration patterns have also been reported by various researchers, noting greater numbers of seedlings, saplings, and mature tree species from the Garhwal Himalaya by Pokhariyal et al. [94], from the katarniaghat Wildlife Sanctuary by Chauhan et al. [95], from the temperate forests of the Kashmir Himalaya by Malik and Bhatt [67], and from the western Himalaya by Haq et al. [96]. The viability of a community is significantly influenced by its ability to regenerate under various environmental factors, including climate, soil properties, disturbance patterns, and seed bank composition [97]. However, some species, such as *Pistacia chinensis* and *Prunus cornuta*, exhibit low seedling establishment due to environmental or anthropogenic disturbances, including altitude variations, lopping, stem cutting, and litter collection [61]. The subalpine zone showed a distinct pattern, with lower seedling and sapling densities compared to mature trees, indicating recruitment challenges. The subalpine zone exhibits lower densities of seedlings and saplings than mature trees, suggesting recruitment challenges stemming from harsh climatic conditions, nutrient-poor soils, biotic factors, limited seed dispersal, reduced pollination efficiency, and anthropogenic pressures [72]. Climate change may further impact forest composition and structure, highlighting the need for conservation efforts [4]. The subtropical zone demonstrated higher regeneration potential for several species, while the temperate zone exhibited moderate success. However, the subalpine zone showed weaker regeneration potential, with limited seedling recruitment for key species such as *Betula utilis*,* Corylus colurna*, and *Quercus semecarpifolia*, raising concerns about long-term population sustainability. In response to these challenges, we proposed long-term monitoring and conservation of tree species diversity in the western Himalayan region of Kashmir, involving local communities that depend on these natural resources for the ecological resilience of the diverse forest ecosystem in the region.

## Conclusion

This study provides valuable data on tree diversity, regeneration patterns, and the influence of human activities in the Western Himalayas. The results show considerable differences in species richness and diversity indices, with the temperate zone having the highest level of biodiversity, followed by the subtropical and subalpine zones. The study also shows that human activities, particularly deforestation and settlements, are important drivers of changes in species composition, particularly in the subtropical and temperate regions. These effects result in changes in tree density, regeneration patterns, and the broader structure of forest ecosystems. Elevation has a significant impact on tree diversity and regeneration, with the subtropical and temperate zones having the most regeneration. The findings highlight the significance of addressing human-caused threats as well as developing long-term forest management measures to mitigate their consequences. Sustainable forest management is crucial for ecosystem services, climate resilience, and biodiversity conservation. This study examines tree diversity and regeneration performance in the western Himalayas, focusing on their distribution and the anthropogenic threats they face. In the subtropical zone, dominated by *Pinus roxburghii*, tree species exhibit limited regeneration due to ecological constraints, soil degradation, and invasive species. Conversely, the temperate zone, known for its high tree diversity, demonstrates good regeneration among coniferous species. However, broadleaf species struggle because of human disturbances, competition, and changing microclimatic conditions. Regenerative forests are vital for restoring degraded ecosystems and facilitating the recolonization of native tree species. The subalpine zone encounters challenge due to harsh climate, limited soil nutrients, and extreme weather conditions. Future conservation initiatives should adopt a zone-specific approach that integrates sustainable management, habitat restoration, and climate adaptation methods. Additionally, public awareness and outreach programs are essential for long-term conservation and sustainable resource utilization. By combining ecological data with management practices, the study presents a framework for ensuring forests’ long-term health and resilience in the face of climate change and human interference.

## Electronic supplementary material

Below is the link to the electronic supplementary material.

Supplementary Material 1

## Data Availability

Data is contained within the article.
